# Prevention and treatment of bleomycin-induced pulmonary fibrosis with the lactate dehydrogenase inhibitor gossypol

**DOI:** 10.1371/journal.pone.0197936

**Published:** 2018-05-24

**Authors:** Jennifer L. Judge, David J. Nagel, Kristina M. Owens, Ashley Rackow, Richard P. Phipps, Patricia J. Sime, R. M. Kottmann

**Affiliations:** 1 Department of Environmental Medicine, University of Rochester, Rochester, NY, United States of America; 2 Lung Biology and Disease Program, University of Rochester, Rochester, NY, United States of America; 3 Department of Medicine, Pulmonary and Critical Care Medicine, University of Rochester, Rochester, NY, United States of America; Centre National de la Recherche Scientifique, FRANCE

## Abstract

Pulmonary fibrosis is a chronic and irreversible scarring disease in the lung with poor prognosis. Few therapies are available; therefore it is critical to identify new therapeutic targets. Our lab has previously identified the enzyme lactate dehydrogenase-A (LDHA) as a potential therapeutic target in pulmonary fibrosis. We found increases in LDHA protein and its metabolic product, lactate, in patients with idiopathic pulmonary fibrosis (IPF). Importantly, we described lactate as a novel pro-fibrotic mediator by acidifying the extracellular space, and activating latent transforming growth factor beta (TGF-β1) in a pH-dependent manner. We propose a pro-fibrotic feed-forward loop by which LDHA produces lactate, lactate decreases pH in the extracellular space and activates TGF-β1 which can further perpetuate fibrotic signaling. Our previous work also demonstrates that the LDHA inhibitor gossypol inhibits TGF-β1-induced myofibroblast differentiation and collagen production *in vitro*. Here, we employed a mouse model of bleomycin-induced pulmonary fibrosis to test whether gossypol inhibits pulmonary fibrosis *in vivo*. We found that gossypol dose-dependently inhibits bleomycin-induced collagen accumulation and TGF-β1 activation in mouse lungs when treatment is started on the same day as bleomycin administration. Importantly, gossypol was also effective at treating collagen accumulation when delayed 7 days following bleomycin. Our results demonstrate that inhibition of LDHA with the inhibitor gossypol is effective at both preventing and treating bleomycin-induced pulmonary fibrosis, and suggests that LDHA may be a potential therapeutic target for pulmonary fibrosis.

## Introduction

Pulmonary fibrosis is a progressive, incurable interstitial lung disease. The median survival from the time of diagnosis is 2.9 years, though the prognosis varies substantially among individuals [1]. There are currently few treatments available for pulmonary fibrosis. Therefore, identification of new therapeutic targets is of critical importance.

The pathogenesis of pulmonary fibrosis remains poorly understood. However, the differentiation of fibroblasts to myofibroblasts is fundamental to the development and progression of fibrosis [2]. Myofibroblasts are one of the key cells in fibrotic lung tissue that are responsible for the production of extracellular matrix proteins such as collagen and fibronectin [3–5]. Agents that target myofibroblast differentiation represent potential therapies for pulmonary fibrosis.

Myofibroblast differentiation is most powerfully induced by the cytokine transforming growth factor-beta (TGF-β1) [6–8]. Furthermore, TGF-β1 induces the production of extracellular matrix proteins as well as contractile smooth muscle proteins such as alpha smooth muscle actin (αSMA) [9,10]. The increased expression of matrix proteins and enhanced contractile properties of myofibroblasts contribute to the progressive restrictive lung disease and diffusion impairment in patients with pulmonary fibrosis. Inhibition of these cellular processes may therefore mitigate the progression of pulmonary fibrosis.

TGF-β1 is produced as a latent protein that requires activation via separation from the latency associated peptide (LAP) [11]. TGF-β1 activation is known to be precipitated by changes in temperature and pH, enzymatic degradation of the LAP, cell surface associations with alpha integrins, and mechanical strain [12–16]. We have previously shown that *in vitro* production of lactic acid by fibroblasts results in physiologic decreases in extracellular pH, which subsequently activate latent TGF-β1 [17]. We have also demonstrated that the enzyme responsible for the production of lactic acid, lactate dehydrogenase-A (LDHA), is elevated in lung tissue isolated from patients with idiopathic pulmonary fibrosis (IPF). In addition, *in vitro* overexpression of LDHA in primary human lung fibroblasts induces activation of TGF-β1 and myofibroblast differentiation [17]. Lastly, we have shown that genetic or pharmacologic inhibition of LDHA inhibits TGF-β1 induced myofibroblast differentiation *in vitro* [18]. Here we hypothesize that pharmacologic inhibition of LDHA via the LDH inhibitor, gossypol [19,20], will abrogate fibrotic changes in an *in* vivo model of bleomycin-induced pulmonary fibrosis. Gossypol is a small molecule inhibitor derived from cottonseed oil [21,22]. We demonstrate that gossypol is effective at inhibiting and treating bleomycin-induced pulmonary fibrosis.

## Materials and methods

### Ethics statement

All animal procedures were approved and supervised by the University of Rochester University Committee on Animal Resources (D16-00188 (A3292-01). Prior to oropharyngeal aspiration procedures, mice were anesthetized with isoflurane. Following oropharyngeal aspiration, mice were monitored on a daily basis to ensure they displayed no evidence of distress. Prior to surgical isolation of the lungs for analysis, mice were anesthetized with an I.P. injection 100 mg/kg ketamine and 10 mg/kg xylazine, followed by exsanguination; all efforts were made to minimize suffering.

### Mice and gossypol dosing

Strict guidelines from the committee were followed regarding the care and handling of mice, anesthesia, and euthanasia in an effort to alleviate suffering. Isoflurane anesthesia was utilized immediately prior to oropharyngeal aspiration and prior to euthanasia. Six to eight week old male C57BL/6J mice were obtained from Jackson Laboratories (Bar Harbor, ME). Mice were dosed with 1.5 U/kg bleomycin (Hospira, Lake Forrest, IL) by oropharyngeal aspiration as previously described to induce fibrosis [23]. The LDH inhibitor gossypol (Sigma Aldrich, St. Louis, MO) was prepared in DMSO and administered by daily sub-cutaneous injection to mice at indicated times at a dose of 5, 10, or 20 mg/kg in a 30μl volume. Mice were euthanized at 4, 7, 14, or 21 days post bleomycin administration. The left lung lobe was inflated and fixed in formalin, the right middle lobe was saved for RNA isolation, and the remaining right lobes were saved for hydroxyproline and protein expression analysis.

### Quantitative real-time polymerase chain reaction

Total RNA was isolated from mouse right middle lung lobes using Trizol reagent (Invitrogen, Carlsbad, CA). Reverse transcription was performed using iScript cDNA synthesis kit (Bio-Rad, Hercules, CA). Real-time polymerase chain reaction was performed using SYBR Green (Bio-Rad). The following primer sequences were used: 18S: **F**:GCTTGCTCGCGCTTCCTTACCT **R**:TCACTGTACCGGCCGTGCGTA, Col1α1 **F**:CTGCTGGCAAAGATGGAG **R**:ACCAGGAAGACCCTGGAATC, Col3α1 **F**:AAATGGCATCCCAGGAG **R**:ATCTCGGCCAGGTTCTC, LDHA **F**:TGGCGACTCCAGTGTGCCTG **R**:AGGCACTGTCCACCTGCT, LDHB **F**: AGTCTCCCGTGCATCCTCAA **R**: AGGGTGTCCGCACTCTTCCT

### Lung tissue histology

At euthanasia, the left lung lobe was inflated and fixed with formalin. Staining was performed on 5 μm thick tissue sections. Trichrome staining for collagen fibers was performed as previously described using Gomori Trichrome reagent (Richard Allen, Thermo Scientific, Pittsburgh, PA)[23]. The histology slides were scored for fibrosis in a blinded manner on the scale of 0 (minimum) to 4 (maximum) as described previously[24]. Immunohistochemistry was performed as previously described using antibodies to LDHA (Abcam, Cambridge, MA), alpha smooth muscle actin (Sigma Aldrich), and fibronectin (Sigma Aldrich) [17], and active TGF-β (R&D Systems, Minneapolis, MN) [25]. Primary antibodies were incubated overnight, followed by washing and addition of a biotinylated secondary antibody (Jackson ImmunoResearch, West Grove, PA) for 1 hour. Streptavidin-HRP (Jackson ImmunoResearch) was added to slides for 15 min, followed by developing with Nova Red (Vector, Burlingame, CA). Slides were counterstained with Hematoxylin (BioCare Medical, Concord, CA) and a coverslip was mounted.

### Hydroxyproline assay

Hydroxyproline content was measured in right lung homogenates using a modified Woessner method as previously reported [23]. Briefly, lung tissue homogenates were hydrolyzed overnight in 6N HCl at 110°C. Samples were neutralized with NaOH, chloramine T reagent was added for 20 minutes, followed by inactivation with 3.15 N perchloric acid. Ehlrich’s solution was added, and samples were incubated for 20 minutes at 60°C. Absorbance was measured at 560 nm and a standard curve using purified hydroxyproline (Sigma Aldrich) was used to extrapolate hydroxyproline content.

### Bronchoalveolar lavage fluid collection, cell counts, and TGF-β1 ELISA

Bronchoalveolar lavage fluid (BALF) was collected at the time of euthanasia by tying off the right lung lobes, instilling PBS into the left lobe, and aspirating the fluid containing protein and cells lining the airways. Cells were pelleted by centrifugation. Supernatant (BAL fluid) was collected for other analyses. Cell pellets were resuspended, red blood cells were lysed, and total cells were counted using a hemocytometer. Differential cell counts were performed with a maximum of 50,000 cells on Cytospin-prepared slides (Thermo Shandon, Pittsburgh, PA). Slides were stained with Diff-Quik (Richard Allen, Thermo Scientific) for differential counting. Cell differentials are reported as a percent of total BAL cells. The remaining BAL fluid was collected and assayed for total TGF-β1 by ELISA (R&D systems, Minneapolis, MN) according to the manufacturer’s instructions.

### Western blots

Western blots were performed using right lung lobe homogenates were performed as previously described [26]. PVDF membranes were probed using the following antibodies: fibronectin (Sigma Aldrich) and β-tubulin (Abcam) and a goat anti-rabbit secondary antibody (Jackson ImmunoResearch). Densitometry was performed using Image Studio Lite software (LI-COR, Lincoln, Nebraska).

### Mass spectroscopy

Standards were prepared from lactic acid (Sigma-Aldrich) and diluted with a 50% mix of HPLC grade methanol in water. Blanks were 50% HPLC grade methanol and water. Lung homogenate samples were diluted 1:1 with a 50% methanol and 50% water to total a volume of 200 μL. Samples were spun at 16,000 rpm at 4°C for 10 minutes. Supernatants were removed and placed in capped vials for mass spectroscopy analysis using the lactate protocol previously described for LC-MS/MS[27].

### LDH activity

Lung tissue was homogenized in PBS and subsequently incubated in the presence of the working buffer containing 0.05 M potassium phosphate pH 7.5, 30 mM pyruvic acid, and 0.25 μM β-NADH. The LDH activity was assessed using spectrophotometry using the millimolar extinction coefficient of NADH of 6.33[28].

## Results

### Lactate dehydrogenase-A is elevated in the lung tissue of mice exposed to bleomycin

**Fig 1:**
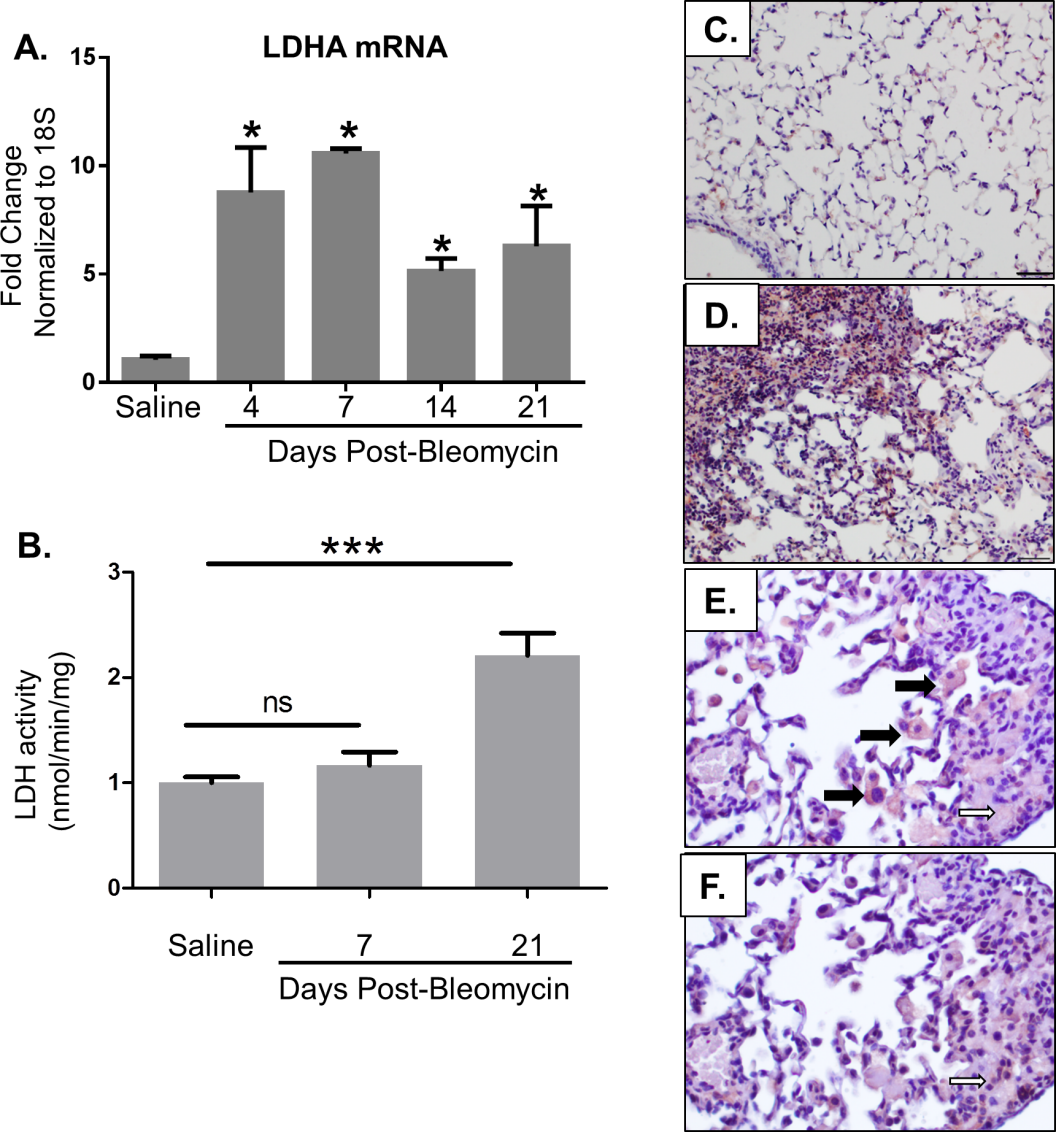
LDHA is increased in bleomycin-induced pulmonary fibrosis. Mice were exposed to saline or bleomycin via oropharyngeal aspiration (OA) and euthanized at indicated time points. (A) Total RNA was isolated from the right lung lobes and qRT-PCR was performed to measure LDHA mRNA. Data are displayed as fold change from saline controls normalized to 18S. *p≤0.05 compared to saline controls by t-test. n = 4–5 per group. (B) LDH activity was measured on lung tissue homogenates in mice administered either saline or bleomycin. ***p<0.001. (C-D) Lung tissue from mice at day 21 post bleomycin was stained for LDHA by immuno-histochemistry in red. Images were taken at 20X magnification, scale bars represent 50 μm. (E-F) Serial section immunohistochemistry was performed for LDH shown in panel E and αSMA shown in panel F. Images were taken at 40X magnification. LDHA expression localized to both macrophages (bold arrows) and areas co-staining for αSMA (open arrows), a marker of myofibroblast differentiation.

We previously discovered that the enzyme lactate dehydrogenase-A (LDHA) is elevated in the lung tissue of patients with IPF [17]. To determine whether LDHA is also increased in the bleomycin-induced pulmonary fibrosis model, mice were exposed to bleomycin or saline by oropharyngeal aspiration. Mice were euthanized on days 4, 7, 14, and 21 post bleomycin. LDHA and B mRNA expression were determined by qRT-PCR (Fig 1A). LDH activity was measured in lung homogenates using a standardized assay (Fig 1B). LDHA protein expression was examined by immunohistochemical staining on lung tissue sections (Fig 1C & 1D). Serial section immunohistochemistry was used to co-localize LDHA expression with αSMA expression, a marker of myofibroblast differentiation. LDHA mRNA was elevated beginning at day 4 post bleomycin, and continued to be elevated until day 21 (Fig 1A). There were no differences in LDHB expression between the groups. LDH activity was increased at day 21 but was not significantly increased at Day7. LDHA protein was increased on day 21 post-bleomycin compared to control lung tissue (Fig 1D). LDH expression localized to both macrophages and the fibrotic interstitium containing αSMA positive cells. Together, these results indicate that bleomycin induces LDHA expression in mouse lung tissue.

### Gossypol prevents bleomycin-induced pulmonary fibrosis

**Fig 2:**
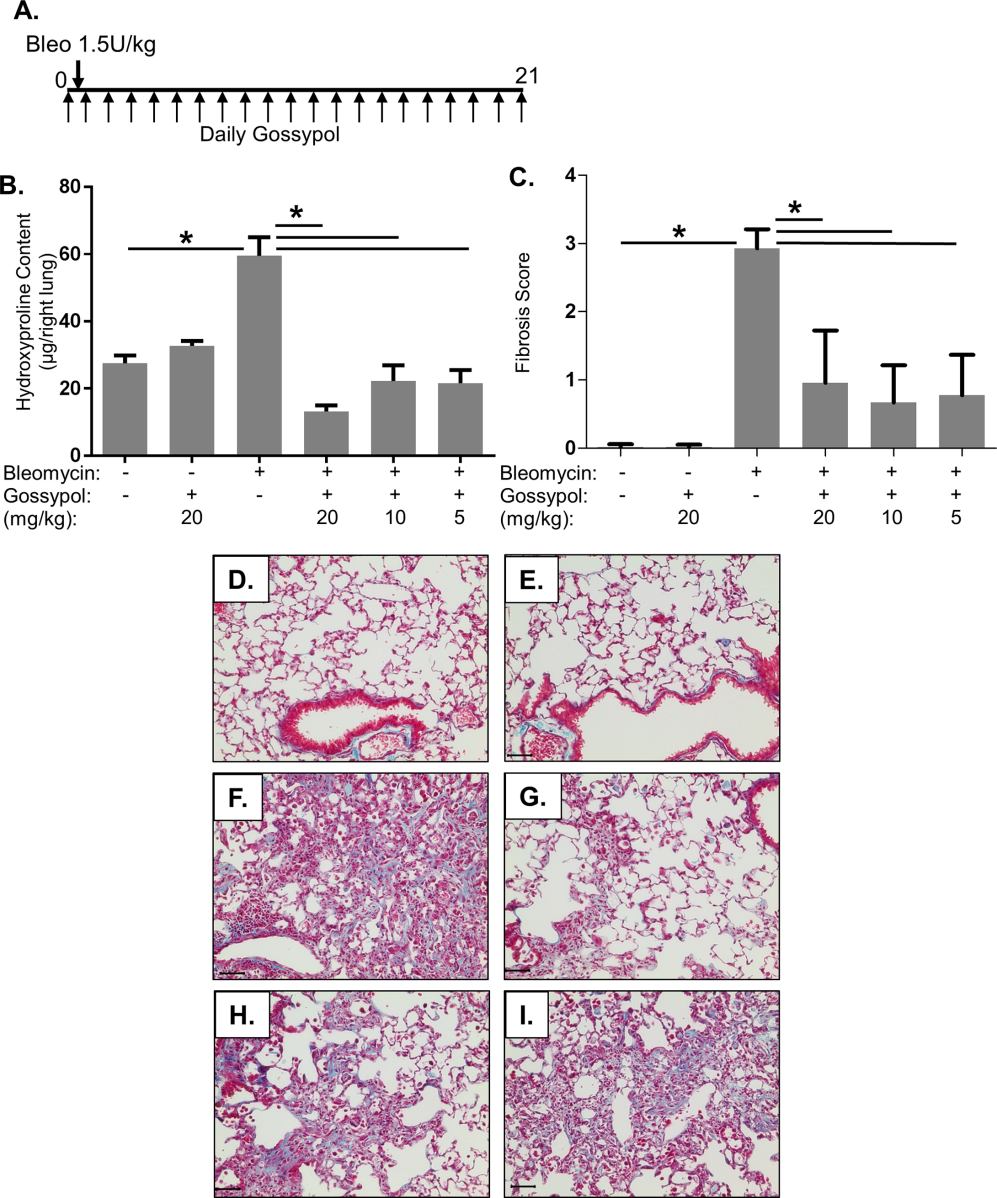
Gossypol prevents bleomycin-induced pulmonary fibrosis. (A) Mice were exposed to 1.5U/kg bleomycin via OA and treated daily with sub-cutaneous injections of the LDHA inhibitor gossypol at 5, 10, or 20 mg/kg until euthanasia at day 21. (B) Collagen levels in the right lung lobes were measured by hydroxyproline assay. Data are displayed as mean ± SEM. *p≤0.05 by ANOVA. n = 9–10 mice per group. (C) Fibrosis scores were performed on trichrome stained sections. Data are displayed as mean ± SEM. *p≤0.05 by ANOVA (D-H) Lung tissue sections were Trichrome stained for collagen fibers in blue. (D) saline + vehicle, (E) saline + gossypol 20mg/kg, (F) bleomycin + vehicle, (G) bleomycin + gossypol 20mg/kg, (H) bleomycin + gossypol 10mg/kg, (I) bleomycin + gossypol 5mg/kg. Images were taken at 20X magnification, scale bar represents 50 μm.

To test whether inhibition of LDHA with the inhibitor gossypol would prevent bleomycin-induced pulmonary fibrosis, mice were exposed to bleomycin or saline by oropharyngeal aspiration (OA), and subsequently dosed with 5, 10, or 20 mg/kg gossypol or vehicle via daily sub-cutaneous injection starting the day of bleomycin administration (Fig 2A). After 21 days, mice were euthanized. Hydroxyproline was measured in right lung lobe homogenates as a measure of collagen content. Bleomycin significantly increased hydroxyproline compared to saline controls (Fig 2B). All three doses of gossypol significantly reduced bleomycin-induced hydroxyproline content, with the greatest effect seen with 20mg/kg (Fig 2B). To visualize fibrosis and collagen accumulation, trichrome staining was performed. Fibrosis scores are shown in Fig 2C. Saline and gossypol only treated mice showed normal lung architecture with very few collagen fibers (blue staining) (Fig 2D & 2E). Mice exposed to bleomycin had significant increases in collagen fibers (Fig 2F). However, bleomycin and gossypol treated mice had reduced collagen fibers compared to bleomycin and vehicle treated mice (Fig 2G–2I). All doses of gossypol decreased fibrosis scores and trichrome staining induced by bleomycin. Together, these results indicate that gossypol prevented bleomycin-induced pulmonary fibrosis.

### Gossypol prevents bleomycin-induced collagen gene expression

**Fig 3:**
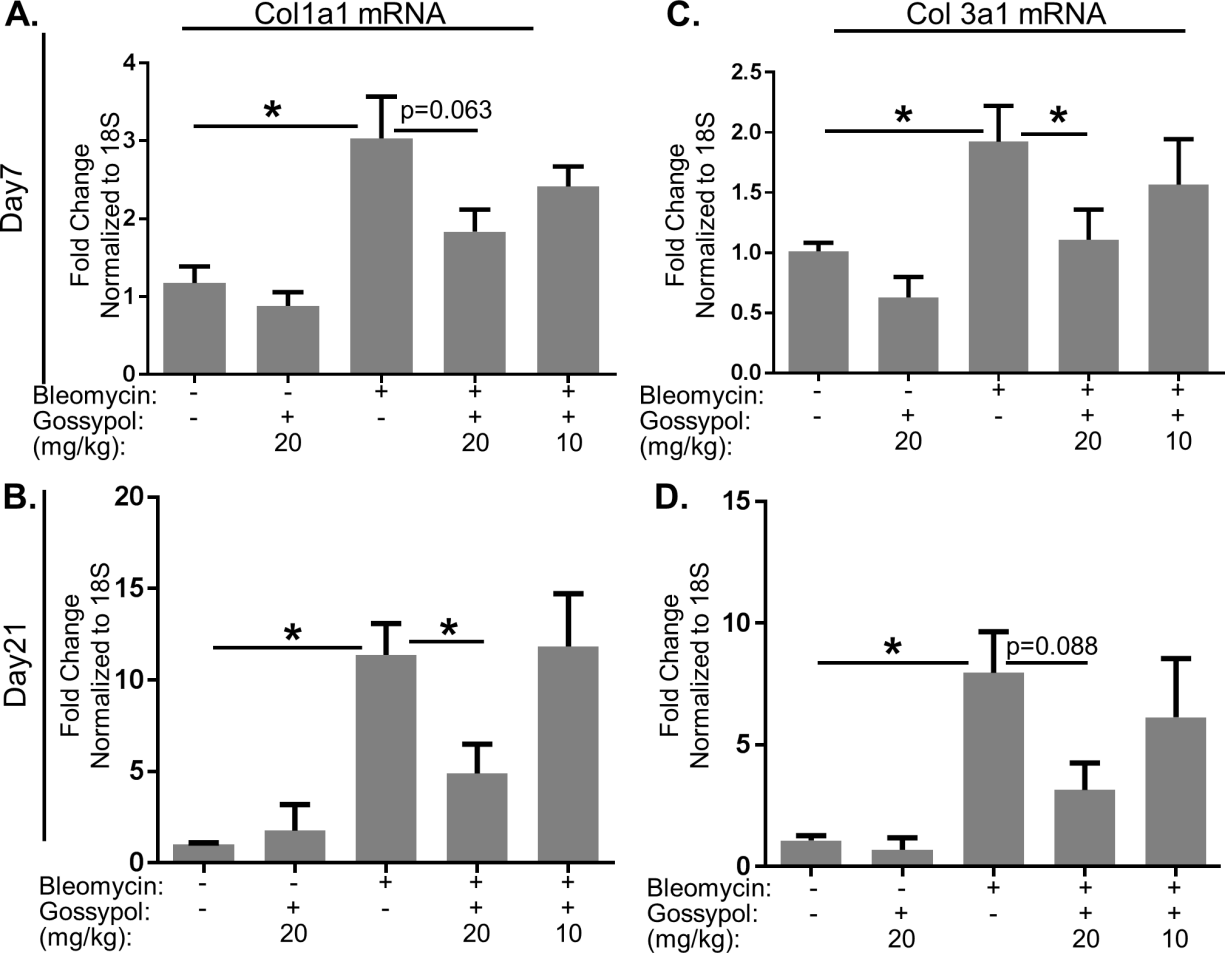
Gossypol prevents collagen gene expression. Mice were exposed to bleomycin and treated with gossypol as indicated in Fig 2 and sacrificed on day 7. Collagen 1α1 and collagen 3α1 gene expression were measured by qRT-PCR. Data are displayed as fold changes from saline and vehicle treated controls normalized to 18S. Collagen 1α1 mRNA was measured on day 7 (A) and day 21 (B). Collagen 3α1 mRNA was measured on day 7(C) and day 21 (D). Data are displayed as mean ± SEM. *p≤0.05 by ANOVA. n = 3–9 mice per group.

To examine whether gossypol regulates bleomycin-induced collagen gene expression, we measured collagen 1 and 3 mRNA expression at 7 and 21 days post-bleomycin. Bleomycin significantly increased collagen 1 and 3 gene expression at days 7 and 21 compared to saline controls (Fig 3). Gossypol reduced bleomycin-induced collagen gene expression in a dose-dependent manner. Collagen 1 gene expression was significantly reduced at day 21 (Fig 3B) while collagen 3 gene expression was significantly reduced at day 7 (Fig 3C) in mice treated with 20mg/kg gossypol. Our results demonstrate that gossypol can regulate collagen at the level of gene expression and may differentially regulate subsets of collagen at different time points over the course of the development of fibrosis.

### Gossypol prevents bleomycin induced expression of fibronectin

**Fig 4:**
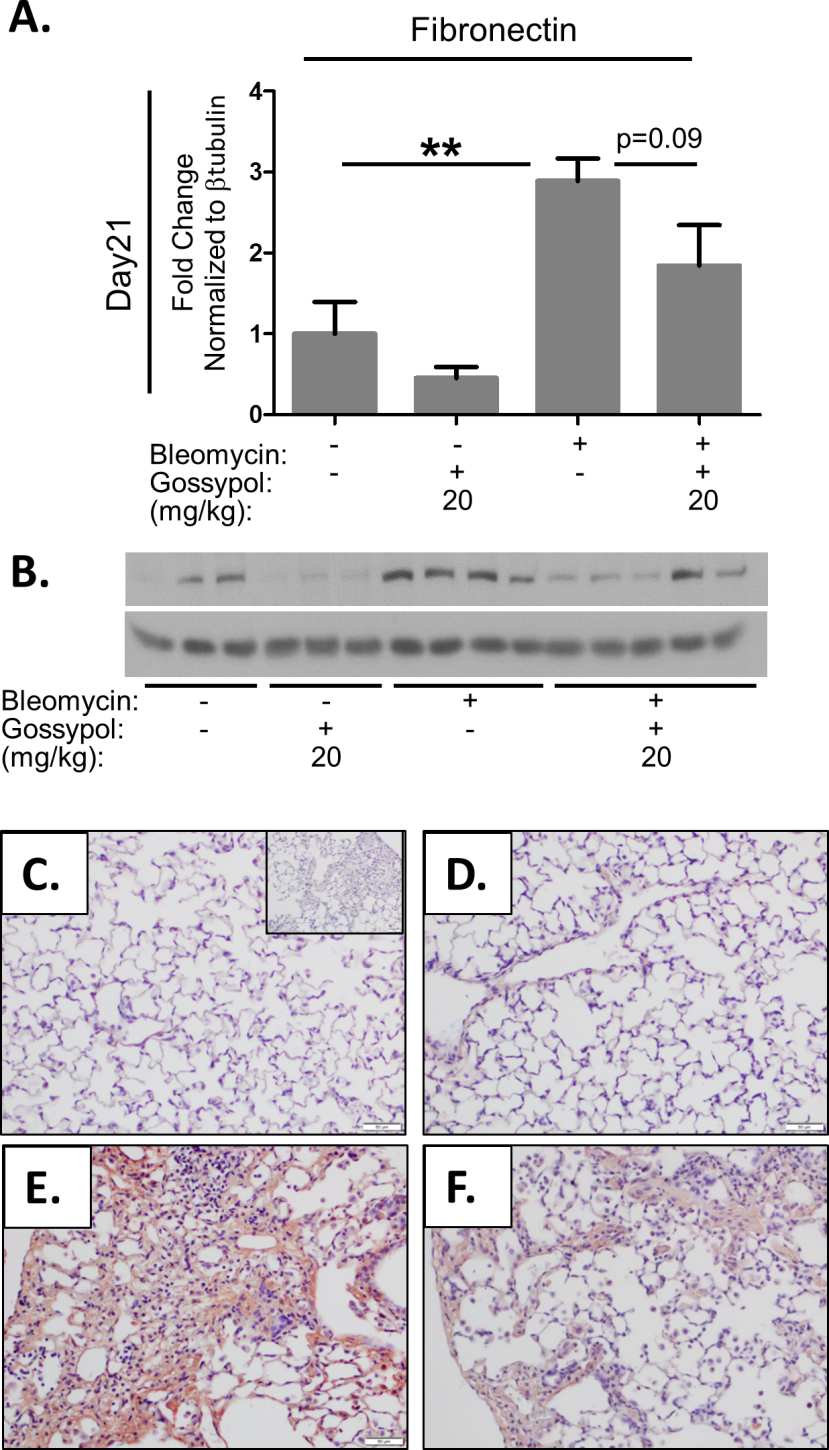
Gossypol prevents TGF-β1 induced fibronectin expression. Mice were exposed to bleomycin and treated with gossypol as indicated in Fig 2 and sacrificed on day 21. Fibronectin expression was measured in whole lung homogenates by Western blot (A) which was quantified using densitometry (B). Data are displayed as mean ± SEM. **p<0.01 by ANOVA. n = 8 mice per treatment group. (C-F) Lung tissue sections were stained for fibronectin in red and counterstained with hematoxylin. (C) Saline + vehicle, (D) saline + gossypol 20mg/kg, (E) Bleomycin + vehicle at (F), bleomycin + gossypol 20mg/kg. All images were taken at 20X magnification (G). Scale bars represent 50 μm. The inset in (C) is an isotype negative control stain.

We next tested whether gossypol would prevent bleomycin induced expression of fibronectin. Fibronectin protein expression was measured by Western blot of lung tissue homogenates and found that mice given bleomycin had significant increase in fibronectin expression compared to controls (Fig 4A and 4B). However, mice that received gossypol following bleomycin had a reduction in expression of fibronectin. We also stained lung tissue sections for fibronectin and found increased fibronectin staining in lungs of mice exposed to bleomycin (Fig 4E) compared to saline (Fig 4C) or gossypol treated controls (Fig 4D). However, mice exposed to bleomycin and dosed with gossypol at 20mg/kg showed less reduced fibronectin staining compared to bleomycin alone, although this did not meet statistical significance (Fig 4F). These data demonstrate gossypol prevents bleomycin induced expression of other matrix proteins involved in the development of pulmonary fibrosis.

### Gossypol prevents LDH Activity and TGF-β1 expression and activation

**Fig 5:**
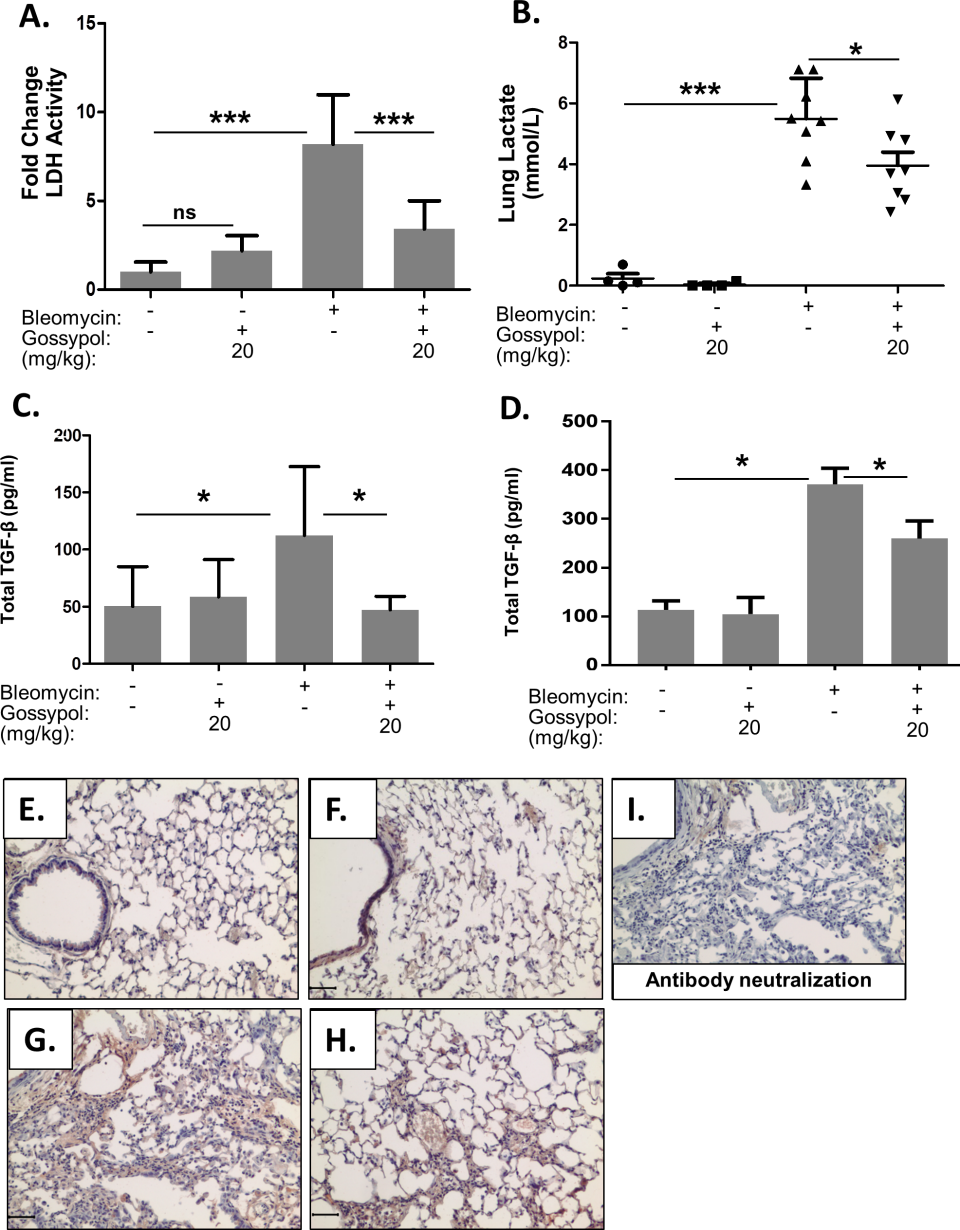
Gossypol inhibits LDH activity and prevents TGF-β1 expression and activation. Mice were exposed to bleomycin and treated with gossypol as indicated in Fig 2. (A) LDH Activity in whole lung homogenates was measured using a standardized protocol. Data are displayed as mean ± SEM. ***p<0.001 by ANOVA. n = 8 mice per treatment. (B) Lactate was measured in whole lung homogenates by mass spectroscopy. Data are displayed as mean ± SEM. ***p<0.001 and *p<0.05 by ANOVA. n = 8 mice per treatment. (C-D) Total TGF-β1 was measured in whole lung tissue homogenates at 7 days (C) and at 21 days (C). *p<0.5 by ANOVA. n = 8 mice per treatment. (E-I) Lung tissue sections were stained for active TGF-β1 by IHC in red and counterstained with hematoxylin. (E) saline + vehicle, (F) saline + gossypol 20mg/kg, (G) bleomycin + vehicle, (H) bleomycin + gossypol 20mg/kg. Antibody neutralization was performed by incubating the primary antibody with human recombinant TGF-β1 for 30 minutes prior to staining to confirm specificity for active TGF-β1 (I). Images were taken at 20X objective, scale bar represents 50 μm.

Transforming growth factor-β1 (TGF- β1) is a critical cytokine that is both necessary and sufficient for the development of pulmonary fibrosis. TGF-β1 must be activated in order to elicit biological effects. We previously reported enhanced LDH activity *in vitro* activates TGF-β1 and induces myofibroblast differentiation[29]. We have also reported that gossypol inhibits LDH activity and myofibroblast differentiation *in vitro*. To determine whether a similar mechanism occurs i*n vivo* we measured LDH activity in whole lung homogenates. Gossypol significantly inhibited bleomycin induced LDH activity at day 21 (Fig 5A). Furthermore, Gossypol also inhibited bleomycin induced whole lung tissue lactate at day 21 (Fig 5B). In order to determine whether Gossypol inhibited the production and activation of TGF-β1, total TGF-β1 was measured in BALF by ELISA. Mice treated with bleomycin had significantly increased levels of TGF-β1 in the BAL fluid compared to vehicle or gossypol-alone treated mice at day 7 (Fig 5C) and at day 21 (Fig 5D). However, mice that were treated with bleomycin and gossypol had significantly reduced levels of total TGF-β1 in the BAL fluid compared to bleomycin and vehicle treated mice, suggesting that gossypol prevented the generation of additional total TGF-β1 protein in lung tissue. Next, we stained for active TGF-β1 on paraffin embedded lung sections. There was very little staining for active TGF-β1 in saline (Fig 5E) and gossypol (Fig 5F) controls. However, mice dosed with bleomycin had increased staining for active TGF-β1, especially in areas of thickened interstitium (Fig 5G). In comparison, mice dosed with bleomycin and treated with gossypol (20mg/kg) had reduced staining for active TGF-β1 (Fig 5H). To confirm specificity for active TGF-β1, we neutralized the primary antibody with human recombinant TGF-β1 for 30 minutes prior to staining. We found very little positive staining after performing the neutralization (Fig 5I).

### Gossypol does not alter inflammatory cell profiles in the lung

**Fig 6:**
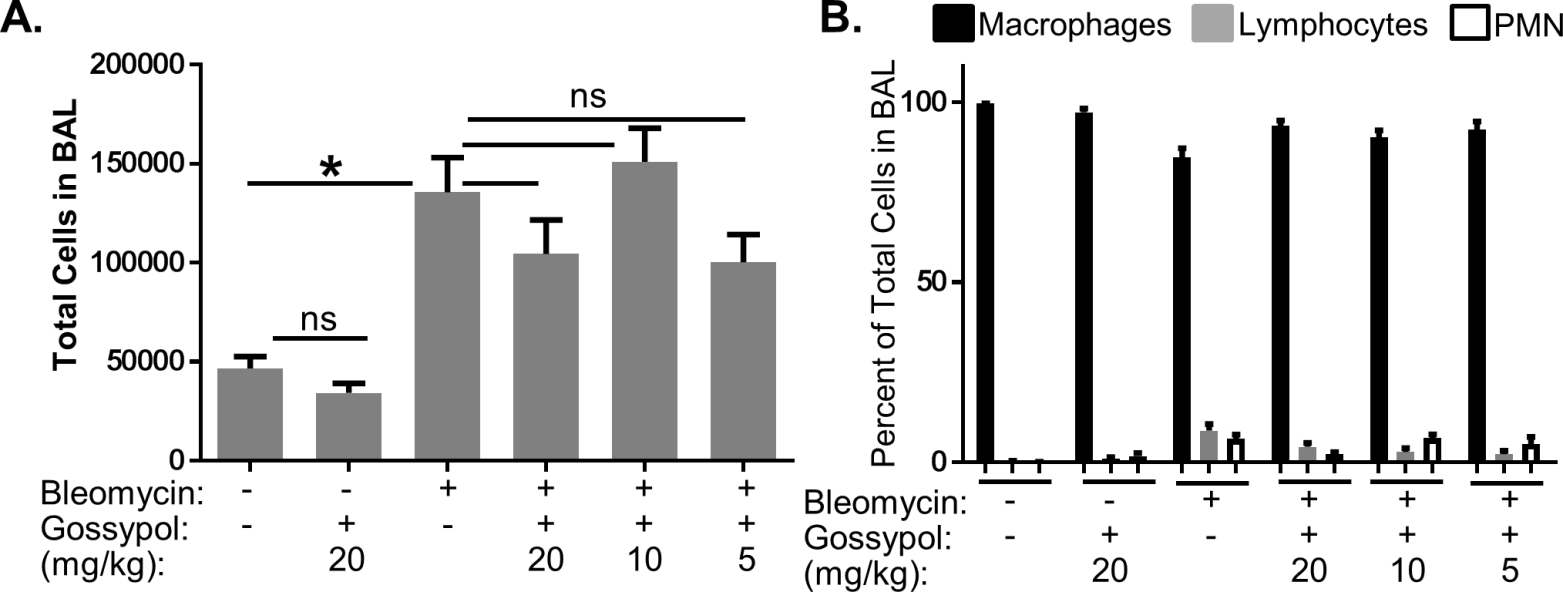
Gossypol does not alter inflammatory cell profiles in the lung. Mice were exposed to bleomycin and treated with gossypol as indicated in Fig 2. Mice were sacrificed on day 7 and the left lung was lavaged with PBS and total cells in lavage fluid were counted (A). Cytospins were prepared, stained with Diff-Quik, and differential counts were performed for macrophages, lymphocytes, and polymorphonuclear (PMN) cells (B). Differential counts are expressed as percent of total cells. Data are displayed as mean ± SEM. *p≤0.05 by ANOVA. n = 5–10 mice.

We next evaluated whether gossypol exhibited anti-inflammatory effects in the lung. Differential cell counts for macrophages, lymphocytes, and polymorphonuclear (PMN) cells in BALF were performed. While bleomycin significantly increased the total number of cells in the BAL fluid (Fig 6A), neither the total cell counts nor differential percentages of cells were not altered by any dose of gossypol (Fig 6B). These data suggest that gossypol inhibits bleomycin-induced fibrosis independent of inflammatory cell profiles in the lung.

### Gossypol is an effective anti-fibrotic treatment strategy

**Fig 7:**
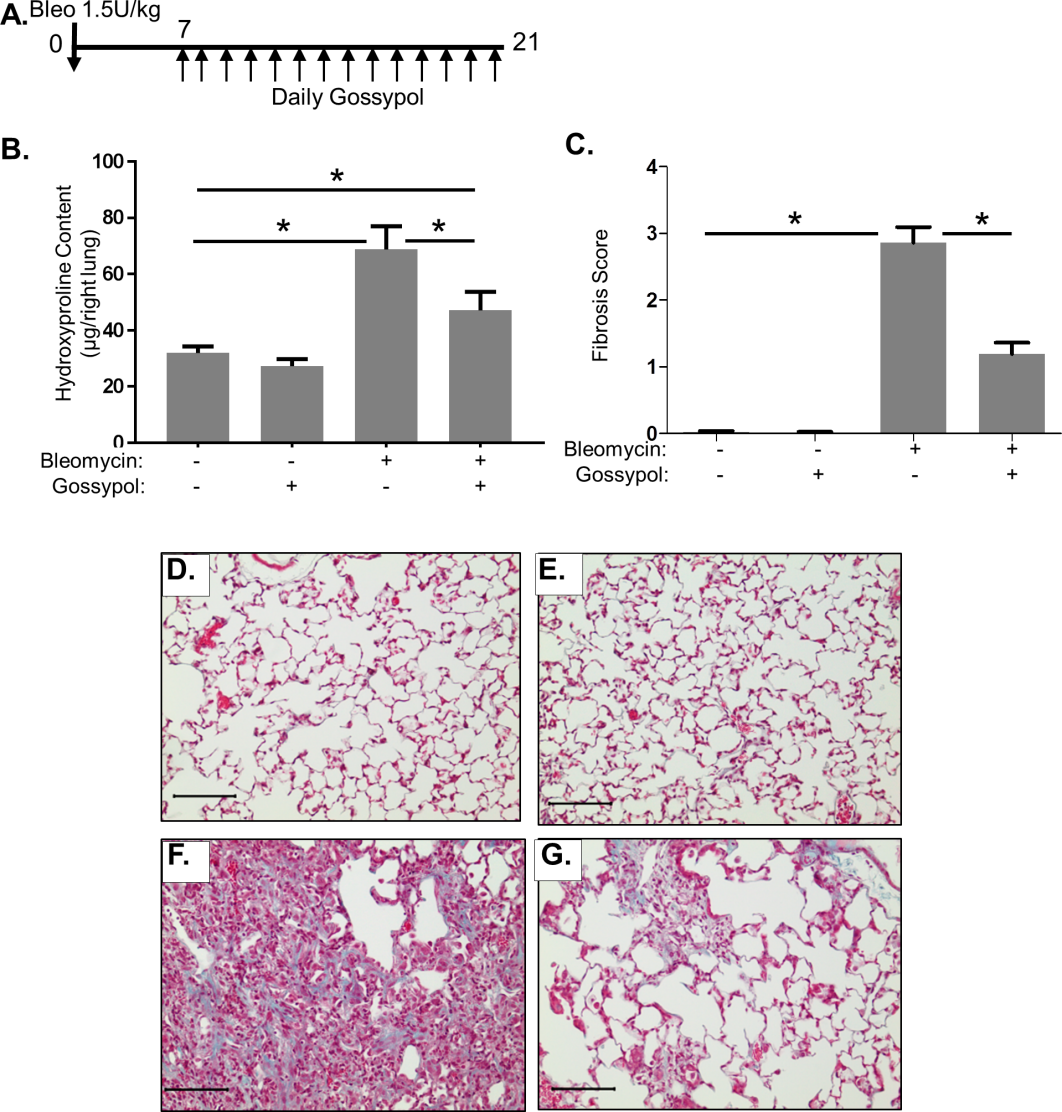
Gossypol is an effective anti-fibrotic treatment. Mice were exposed to 1.5 U/kg bleomycin by OA. (A) Starting at day 7 post-bleomycin, mice were dosed with 20 mg/kg gossypol or vehicle until day of sacrifice at 21 days. (B) Collagen levels in the right lung lobes were measured by hydroxyproline assay. Data are displayed as mean ± SEM. *p≤0.05 by ANOVA. n = 7–10 mice per treatment. (C) Fibrosis scoring was performed on trichrome stained lung tissue sections. (D-F) Collagen fibers were visualized by Trichrome stain in blue. (D) Saline + vehicle, (E) bleomycin + vehicle, (F) saline + gossypol 20mg/kg, (G) bleomycin + gossypol 20mg/kg. Images were taken at 20X objective, scale bar represents 100 μm.

Our results thus far demonstrate that gossypol can prevent bleomycin-induced fibrosis. However, we also wanted to test whether gossypol may be a therapy for pulmonary fibrosis. To examine this, we exposed mice to 1.5U/kg bleomycin but delayed treatment with gossypol until day 7 of the experiment at which point mice were treated with daily subcutaneous gossypol (20mg/kg) until euthanasia at day 21 post-bleomycin (Fig 7A). We assessed fibrosis by a hydroxyproline assay for collagen content, and trichrome staining. Bleomycin significantly increased hydroxyproline content compared to saline and gossypol treated control mice (Fig 7B). However, mice that received gossypol following bleomycin had significantly reduced lung hydroxyproline content compared to bleomycin and vehicle treated mice (Fig 7B). To visualize lung architecture and collagen fibers, we performed trichrome stains on lung tissue sections and sections were scored for extent of fibrosis (Fig 7C). Saline treated mice had normal lung architecture (Fig 7D) and as did gossypol treated controls (Fig 7E). Mice exposed to bleomycin and treated with vehicle showed a significant increase in blue staining for collagen fibers and dense areas of fibrosis (Fig 7F). Mice treated with gossypol showed largely normal lung architecture with fewer areas of collagen fiber accumulation (Fig 7G). Together our findings demonstrate that gossypol is effective at treating existing bleomycin-induced fibrosis.

### Gossypol treats bleomycin-induced fibronectin accumulation

**Fig 8:**
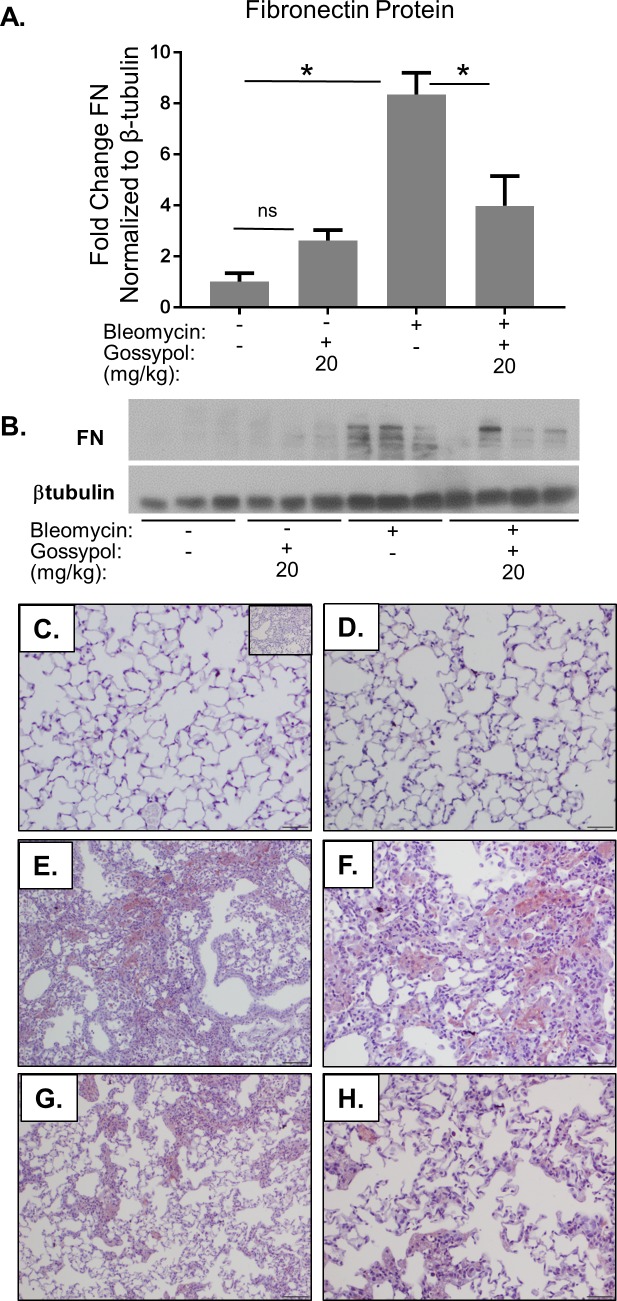
Gossypol treats bleomycin-induced fibronectin expression. Mice were exposed to bleomycin and treated with gossypol as indicated in Fig 7. Lung homogenates were probed for fibronectin protein by western blot and quantified using densitometry (A). One representative blot is shown, each lane represents one mouse (B). Data are displayed as mean ± SEM. *p≤0.05 by ANOVA. n = 3–6 mice per group. (C-H) Lung tissue sections were stained for fibronectin in red and counterstained with hematoxylin. (C) Saline + vehicle, (D) saline + gossypol 20mg/kg, Bleomycin + vehicle at 10X magnification (E) and 20X magnification (F), bleomycin + gossypol 20mg/kg at 10X magnification (G) and 20X magnification (H). Scale bars represent 50 μm. Inset in (C) is an isotype negative control stain.

To examine the effects of gossypol on other extracellular matrix components, we measured levels of fibronectin protein by western blot and found that mice given bleomycin had an eight-fold induction in fibronectin expression compared to controls (Fig 8A and 8B). However, mice that received gossypol following bleomycin had a significant reduction in expression of fibronectin. We again stained lung tissue sections for fibronectin and found increased fibronectin staining in lungs of mice exposed to bleomycin (Fig 8E & 8F) compared to saline (Fig 8C) or gossypol treated controls (Fig 8D). However, mice exposed to bleomycin and dosed with gossypol at 20mg/kg showed significantly reduced fibronectin staining compared to bleomycin alone (Fig 8G & 8H). These data demonstrate that even delayed treatment with gossypol inhibited bleomycin-induced fibronectin accumulation in the lung.

## Discussion

In this manuscript, we demonstrate that the LDH inhibitor, gossypol, prevents and treats *in vivo* bleomycin-induced pulmonary fibrosis. We have shown that LDHA expression is increased in the lung tissue of mice exposed to bleomycin and that the increase in LDHA gene expression occurs within the first several days following bleomycin administration (Fig 1A). An in increase in whole lung tissue LDH activity is not evident at day 7 but is significantly increased at day 21 (Fig 1B). LDHA protein expression is significantly elevated in the lung tissue of bleomycin exposed mice at day 21 (Fig 1D) and co-localizes to regions that stain prominently for αSMA (Fig 1E and 1F). Here we hypothesized that the increase in LDHA expression *in vivo* subsequently induces the development of pulmonary fibrosis. To test this hypothesis, mice exposed to bleomycin were treated with a pharmacologic inhibitor of LDH, gossypol [19,20,30] (Fig 2A). Gossypol inhibited bleomycin-induced pulmonary fibrosis (Fig 2B and 2C–2H), pro-fibrotic gene expression (Fig 3A–3D) and fibronectin expression (Fig 4). Gossypol inhibited LDH activity and lactate accumulation in whole lung homogenates (Fig 5A and 5B) and reduced the bleomycin-induced increase in total TGF-β1 concentrations in BALF at day 7 and day 21 (Fig 5C and 5D). Furthermore, gossypol also significantly reduced the amount of active TGF-β1 expression in lung tissue (Fig 5E and 5H). Gossypol did not affect bleomycin-induced increases in total cell counts in BALF nor did it significantly alter the cellular differential percentages (Fig 6), suggesting that the inhibitory effects on fibrosis are not merely the result of a decrease in bleomycin-induced inflammation. Importantly, we found that gossypol was also effective at treating bleomycin-induced pulmonary fibrosis when treatment was delayed for 7 days after bleomycin administration (Figs 7 and 8). These data suggest that LDHA inhibition is a potential therapeutic strategy for pulmonary fibrosis even when fibrosis is already established.

These *in vivo* data support our prior studies that show that TGF-β1 and LDHA contribute to a pro-fibrotic feed-forward loop whereby TGF-β1 induces expression of LDHA and a reduction in extracellular pH, which subsequently potentiates activation of additional stores of TGF-β1 [17,18,25]. We previously demonstrated the importance of extracellular acidification in myofibroblast differentiation [17,18,25], and have also shown that bleomycin induces a decrease in the pH of mouse lung tissue to 6.8 [31]. Importantly, interruption of this feed-forward loop inhibits TGF-β1 induced myofibroblast differentiation *in vitro* [18]. Here we demonstrate that by inhibiting LDHA with the pharmacologic inhibitor gossypol, results in decreased LDH activity, lactate accumulation and activation of TGF-β1. Although we did not demonstrate differences in LDH activity at early time points during the development of fibrosis, we suspect that LDH activity is increased at these time points but in a regional manner that is below the limit of detection in diluted lung homogenates. Furthermore, we demonstrated that gossypol inhibits bleomycin induced accumulation of lactate in lung tissue. However, the amount of lactate in mice treated with both bleomycin and gossypol was still well above untreated controls. We suspect the persistent increase in lactate may be related to the influx of inflammatory cells including macrophages that infiltrate airways and may not be inhibited to the same degree as interstitial cells in close proximity to the vasculature. Additionally, the total amount of lung lactate may not be the most significant driver of fibrosis but rather the rate at which lactate accumulates in the extracellular space. Gradual lactate accumulation may not affect the lung tissue pH as dramatically and therefore induce less activation of TGF-β1. While we do not have direct evidence that gossypol prevents a bleomycin-induced decreases in lung tissue pH in these studies, our prior *in vitro* studies strongly suggest that rate of acidification is significantly inhibited by gossypol which in turn inhibits activation of TGF-β1. Overall these data further support our proposed pro-fibrotic feed-forward loop involving.

Excess LDHA expression and/or activity have been directly linked to a variety of clinical conditions including Non-Hodgkin Lymphoma [32], melanoma [33], gastric cancer [34], non-small cell cancer [35], and the aging brain [36]. Targeted LDHA inhibition including the use of the pharmacologic inhibitor, gossypol, is currently being studied in several clinical contexts [37–42]. Although genetic silencing of LDHA may someday be plausible for *in vivo* applications, pharmacologic approaches are the most feasible option.

Gossypol is known to promote apoptosis and inhibit cell cycle progression [30,43–45]. While we cannot exclude the contribution of these off target effects, our previous *in vitro* studies demonstrate that both genetic and pharmacologic inhibition of LDHA reduce pH-dependent activation of TGF-β1 [17]. The established role of extracellular pH and its ability to activate latent TGF-β1 along with the observation of similar effects of gossypol *in vitro* and *in vivo* are all consistent with the hypothesis that LDHA inhibition is the primary mechanism by which gossypol inhibits pulmonary fibrosis. Our prior *in vitro* studies using gossypol showed that there was not a significant effect on cell viability and importantly, there were no obvious lung tissue abnormalities in mice that received gossypol (Fig 2). These data suggest that although gossypol can induce apoptosis in cancer cells, it does not significantly induce cell death at doses that prevent and treat bleomycin-induced pulmonary fibrosis.

Other recent studies have implicated metabolic enzymes may be associated with the development of pulmonary fibrosis. Xie *et*. *al*. reported that inhibition of 6-phosphofructo-2-kinase/fructose-2,6-biphosphatase 3 (PFKFB3), a glycolytic enzyme that is upstream of LDHA, also inhibits bleomycin-induced pulmonary fibrosis [46]. However, they did not specifically address whether inhibition of PFKFB3 also inhibited extracellular acidification or activation of TGF-β1. Our lab has also reported the importance of LDH inhibition in radiation induced fibrosis. We previously demonstrated that Gossypol inhibits radiation induced collagen accumulation as well as activation of TGF-β1[25,47]. We hypothesize that LDHA is key regulator of TGF-β1 induced fibrosis via the regulation of extracellular pH. Importantly, LDHA inhibition may be a more appropriate means to indirectly reduce TGF-β1 activation, as direct neutralization presents concerns with immune regulation and deficiencies in wound healing.

Inhibition of LDHA is gaining clinical prominence as a therapeutic intervention for diseases such as cancer and aging [36,38–42]. In our manuscript, we highlight the potential of LDHA inhibitors as a therapy for pulmonary fibrosis. Our data build on previous data in support of the role of LDHA inhibition in the prevention and reversal of TGF-β1 induced myofibroblast differentiation and the subsequent development of pulmonary fibrosis. Although gossypol is currently being studied in phase 1 and 2 clinical trials, it is not yet clear whether this LDHA inhibitor will be effective in the mitigation of human diseases. It may eventually be possible to develop newer generations of LDHA inhibitors that are more potent, target specific isoenzymes or that target specific organs or cell types. Nonetheless, LDHA inhibitors have therapeutic potential and represent a promising novel target for pulmonary fibrosis.

## Supporting information

S1 File 1968_btub_blot_scan.tif. Gossypol treats bleomycin-induced fibronectin expression. Mice were exposed to bleomycin and treated with gossypol as indicated in Fig 7. Lung homogenates were probed for fibronectin and β-tubulin protein by western blot. The uncropped β-tubulin film is shown here.
(TIF)

S2 File 1968 Fn_scanned_blot. Gossypol treats bleomycin-induced fibronectin expression. Mice were exposed to bleomycin and treated with gossypol as indicated in Fig 7. Lung homogenates were probed for fibronectin and β-tubulin protein by western blot. The uncropped fibronectin film is shown here.
(TIF)

S3 File 2513_btub. Gossypol prevents bleomycin-induced fibronectin expression. Mice were exposed to bleomycin and treated with gossypol as indicated in Fig 2. Lung homogenates were probed for fibronectin and β-tubulin protein by western blot. The uncropped β-tubulin film is shown here.
(TIF)

S4 File 2513_Fn. Gossypol prevents bleomycin-induced fibronectin expression. Mice were exposed to bleomycin and treated with gossypol as indicated in Fig 2. Lung homogenates were probed for fibronectin and β-tubulin protein by western blot. The uncropped fibronectin film is shown here.
(TIF)
