# Supplementary figures and images for: Prevention and treatment of bleomycin-induced pulmonary fibrosis with the lactate dehydrogenase inhibitor gossypol
Source: PLoS One. 2018 May 24;13(5):e0197936. doi: 10.1371/journal.pone.0197936 (PMC5967738; doi:10.1371/journal.pone.0197936)

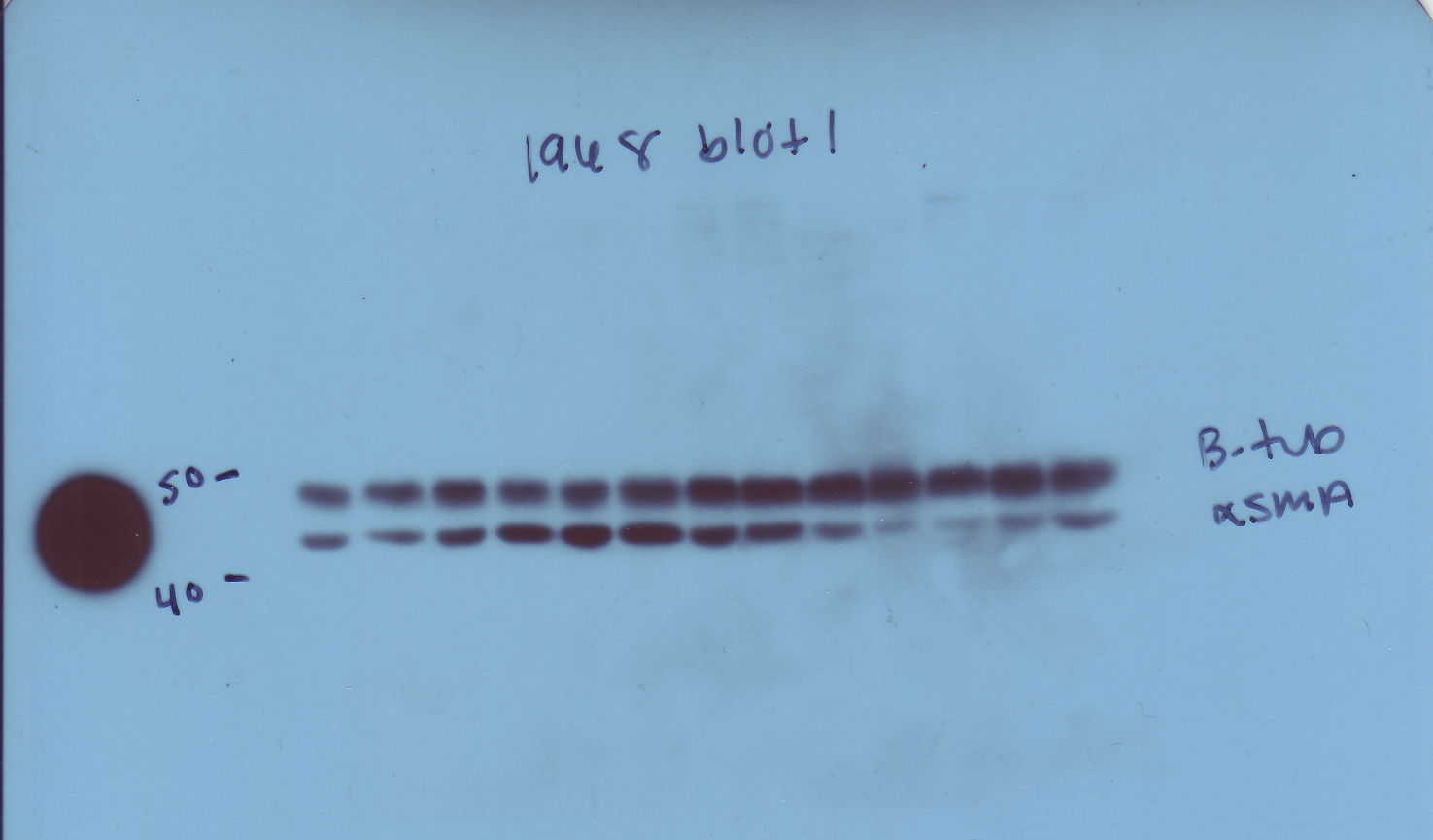

Supplement: S1 File — Gossypol treats bleomycin-induced fibronectin expression. Mice were exposed to bleomycin and treated with gossypol as indicated in Fig 7. Lung homogenates were probed for fibronectin and β-tubulin protein by western blot. The uncropped β-tubulin film is shown here. (TIF) [file pone.0197936.s001.tif]

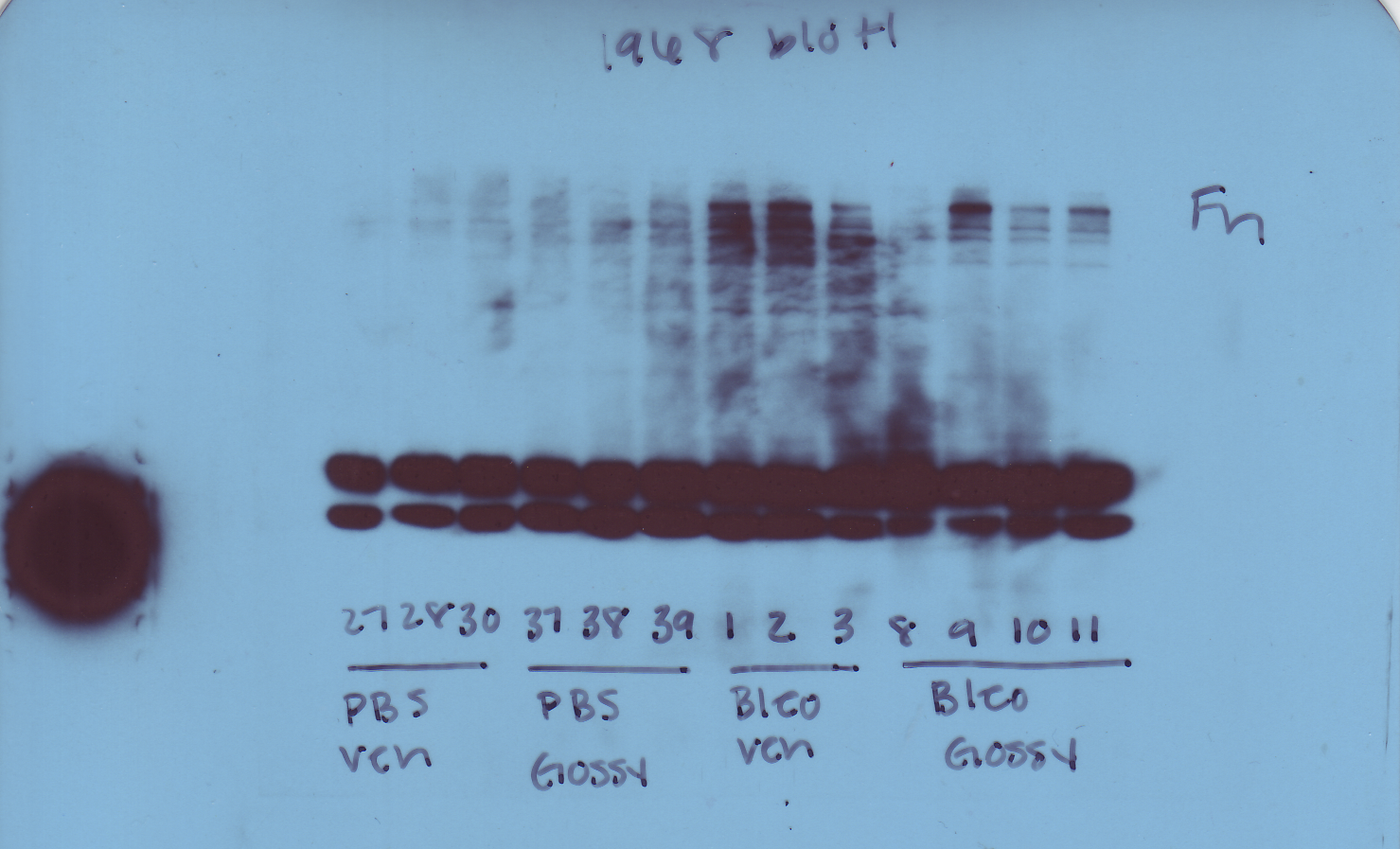

Supplement: S2 File — Gossypol treats bleomycin-induced fibronectin expression. Mice were exposed to bleomycin and treated with gossypol as indicated in Fig 7. Lung homogenates were probed for fibronectin and β-tubulin protein by western blot. The uncropped fibronectin film is shown here. (TIF) [file pone.0197936.s002.tif]

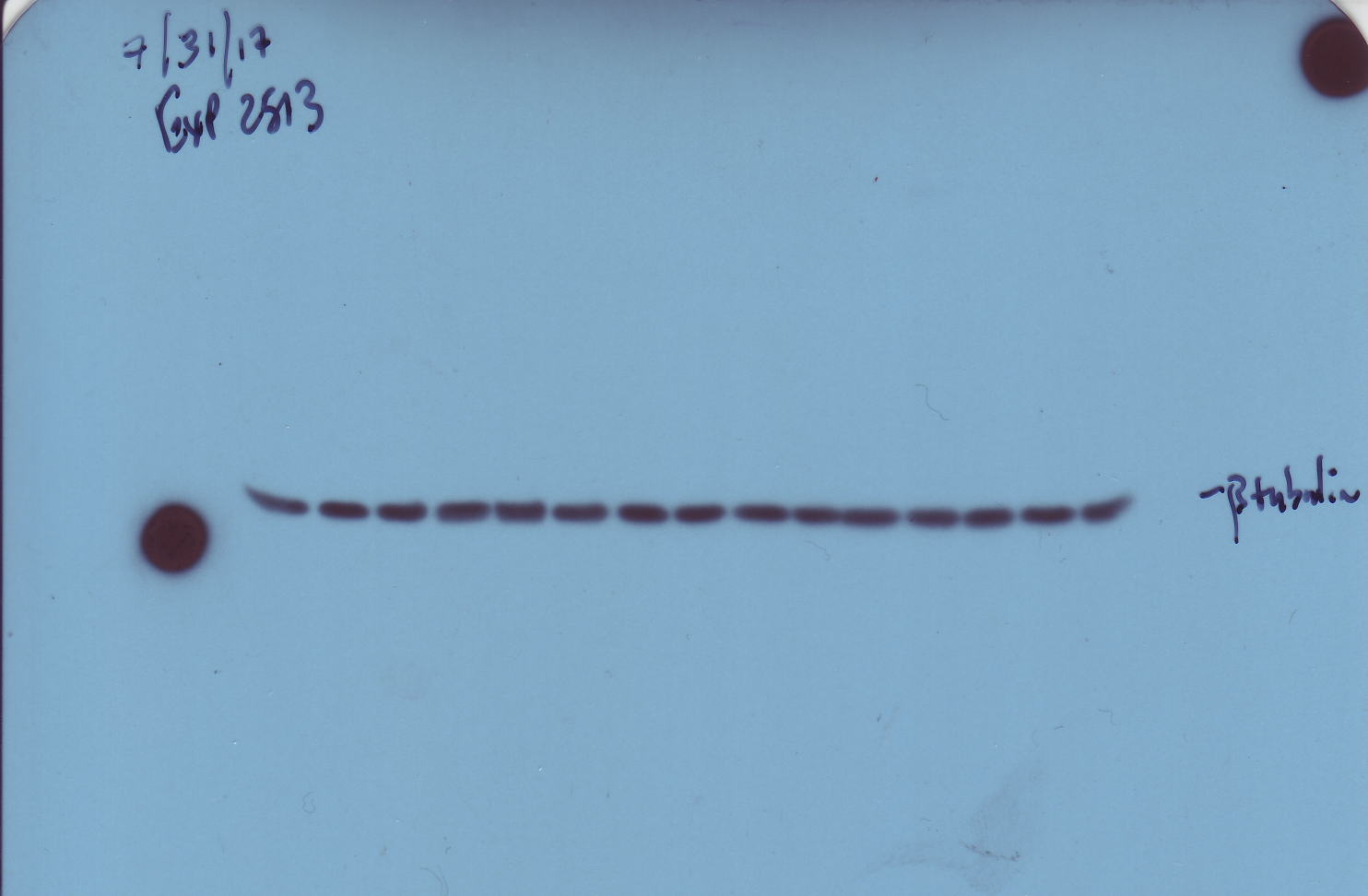

Supplement: S3 File — Gossypol prevents bleomycin-induced fibronectin expression. Mice were exposed to bleomycin and treated with gossypol as indicated in Fig 2. Lung homogenates were probed for fibronectin and β-tubulin protein by western blot. The uncropped β-tubulin film is shown here. (TIF) [file pone.0197936.s003.tif]

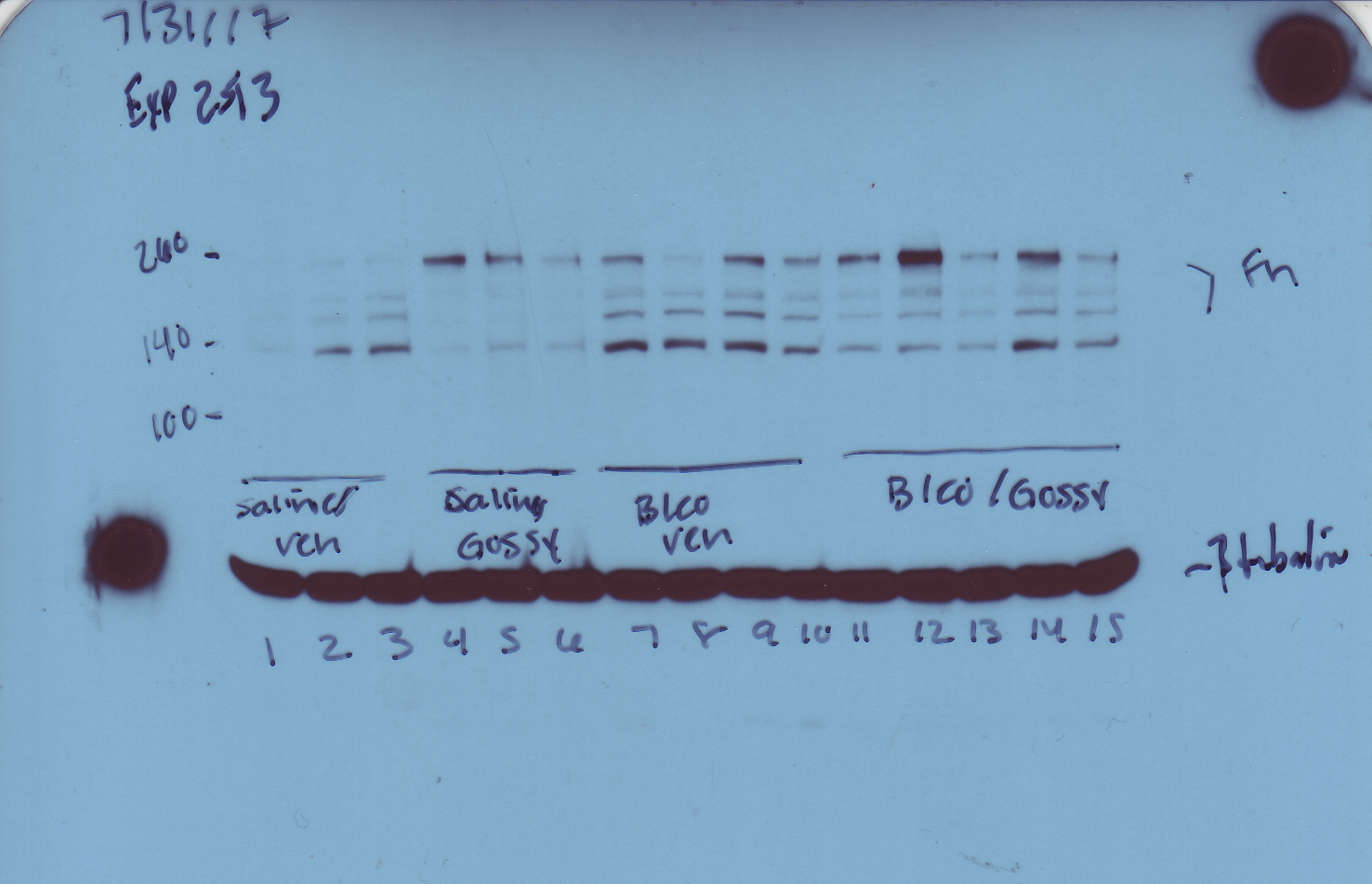

Supplement: S4 File — Gossypol prevents bleomycin-induced fibronectin expression. Mice were exposed to bleomycin and treated with gossypol as indicated in Fig 2. Lung homogenates were probed for fibronectin and β-tubulin protein by western blot. The uncropped fibronectin film is shown here. (TIF) [file pone.0197936.s004.tif]
